# Empirical Model for Evaluating PM_10_ Concentration Caused by River Dust Episodes

**DOI:** 10.3390/ijerph13060553

**Published:** 2016-06-02

**Authors:** Chao-Yuan Lin, Mon-Ling Chiang, Cheng-Yu Lin

**Affiliations:** Department of Soil and Water Conservation, National Chung Hsing University, 250, Kuo-Kuang Rd., Taichung 40227, Taiwan; cylin@water.nchu.edu.tw (C.-Y.L.); ling2652567@yahoo.com.tw (M.-L.C.)

**Keywords:** river dust episode, PM_10_, regression analysis, supervised classification

## Abstract

Around the estuary of the Zhuo-Shui River in Taiwan, the waters recede during the winter, causing an increase in bare land area and exposing a large amount of fine earth and sand particles that were deposited on the riverbed. Observations at the site revealed that when northeastern monsoons blow over bare land without vegetation or water cover, the fine particles are readily lifted by the wind, forming river dust, which greatly endangers the health of nearby residents. Therefore, determining which factors affect river dust and constructing a model to predict river dust concentration are extremely important in the research and development of a prototype warning system for areas at risk of river dust emissions. In this study, the region around the estuary of the Zhuo-Shui River (from the Zi-Qiang Bridge to the Xi-Bin Bridge) was selected as the research area. Data from a nearby air quality monitoring station were used to screen for days with river dust episodes. The relationships between PM_10_ concentration and meteorological factors or bare land area were analyzed at different temporal scales to explore the factors that affect river dust emissions. Study results showed that no single factor alone had adequate power to explain daily average or daily maximum PM_10_ concentration. Stepwise regression analysis of multiple factors showed that the model could not effectively predict daily average PM_10_ concentration, but daily maximum PM_10_ concentration could be predicted by a combination of wind velocity, temperature, and bare land area; the coefficient of determination for this model was 0.67. It was inferred that river dust episodes are caused by the combined effect of multiple factors. In addition, research data also showed a time lag effect between meteorological factors and hourly PM_10_ concentration. This characteristic was applied to the construction of a prediction model, and can be used in an early warning system for local residents.

## 1. Introduction

Particulate matter less than 10 μm in diameter (PM_10_) is defined as respirable particulate matter that can accumulate in the lungs and is harmful to human health [1]. Monitoring and forecasting PM_10_ pollution platforms should be developed to inform the public of harmful air pollution events, as well as to adapt air pollution control strategies [2,3]. Deterministic and statistical models are the two main approaches to the prediction of PM_10_ [3]. The complexity of deterministic approaches, which require detailed information on physical–chemical processes in the atmosphere and detailed knowledge concerning the emission sources of various pollutants, could lead to inaccurate PM_10_ prediction for short and medium ranges [2,4]. The detailed emission data and other parameters are generally limited, thus the parameters should be estimated or simply ignored, which eventually increases the uncertainty of the results [5]. Under certain conditions, statistical models or empirical models, due to their simplicity, are alternative approaches that are frequently used to predict PM_10_ for complex site-specific solutions with acceptable accuracy [2,3,6,7,8]. Predictions of PM_10_ concentrations based on related meteorological parameters are reported in the literature [9,10,11]. In order to improve the accuracy of PM_10_ estimation, related parameters such as satellite-derived parameters and land use/land cover data are also used to develop more complex models, such as multiple regressive and nonlinear models [12,13].

One of the PM_10_ emission sources is aeolian dust, which is associated with the wind erosion of soils [14]. Dust particles originate from several different sources e.g., deserts, coastal areas, agricultural areas, alluvial fans, dry lake beds, and dry river beds [15]. In Taiwan, river dust episodes most frequently occur during the winter when northeastern monsoons are prevalent, and most seriously affect residents and agricultural production near rivers and estuaries [16,17,18,19]. After the last major precipitation event in the fall, low rainfalls during winter droughts cause river waters to recede. As the moisture content of the riverbed soil decreases, the sand, silt, and clay particles that were transported downstream and deposited on the riverbed surface become a primary source of fugitive dust. In addition, transverse structures such as water dams or check dams have been built across the upper reaches of some rivers in Central Taiwan, preventing sizable quantities of water and larger earth and sand particles from moving downstream. This has caused an increase in the area of exposed bare riverbed as well as a decrease in particle diameter distribution, greatly increasing the likelihood of a river dust episode [20]. By processing data from the air quality monitoring station at Lun-Bei, analyzing the chemical composition of the aerosol, and receptor modeling, it was determined that PM_10_ concentrations at the Lun-Bei station have increased substantially since 2004, and that conditions are worst at the estuary of the Zhuo-Shui River near Lun-Bei. For these reasons, factors that affected river dust emission near the estuary of the Zhuo-Shui River were explored in this study.

Numerous factors can cause river dust emissions, but one of the key reasons is wind velocity [21]. The magnitude of wind velocity often determines the amount of wind erosion and sediment movement patterns. After the wind erosion of a bare riverbed, the fine sediment particles that originally settled on the riverbed are lifted by the wind and become the primary source of PM_10_. Therefore, a higher wind velocity may result in a higher PM_10_ level [22,23]. Temperature affects air convection and water vapor saturation. Differences in temperature can cause convection of land and sea breezes, rising and falling air currents, and air currents from local differences in land cover. These indirectly affect the transport of particles suspended in the air. In addition, diurnal and seasonal temperature variations affect water vapor saturation in the air. Higher temperatures cause more evaporation, which can produce cracks in a bare riverbed. In combination with sufficient wind velocity, this can result in wind erosion. Thus, temperature is one of the factors that effect variations in PM_10_ [24]. Humidity is related to the amount of water vapor in the air. Although temperature limits the saturation pressure of water vapor, factors such as seasonal and regional variations or rainfall can produce variations in humidity and affect the transport of suspended particles. Thus, humidity is also considered one of the meteorological factors that affects river dust episodes [25,26].

In addition to meteorological factors, other important factors are the amount of bare land and soil moisture. A greater area of bare riverbed represents a greater amount of source materials for river dust [27,28]. Chen *et al.* [29] explored the relationship between soil moisture and threshold velocity for soil particle movement by wind using a wind tunnel experiment. However, the evaluation of bare land area and soil moisture derived from field surveys is time-consuming. Several studies applied Aerosol Optical Thickness (AOT) extracted from MODerate resolution Imaging Spectro-radiometer (MODIS) to estimate PM_10_ concentration [1,9,10,12,13,30] but the low resolution of MODIS (1 × 1 km^2^) is not suitable for estimating local river dust. Many studies have developed land-use regression (LUR) for air pollution mapping and have applied it to a large number of unsampled locations [31]. Although the performance of this method is generally good and remarkably consistent in different study areas [32], very few studies consider the impact of land use/land cover change (LUCC), which is a key factor influencing atmospheric particulate pollution [33]. In addition, the LUR method is suitable for monitoring sites from a long-term spatio-temporal perspective [34], whereas this study aimed to predict PM_10_ from site-specific river dust episodes.

During river dust episodes, the effect of land cover changes and soil moisture could result in PM_10_ prediction. Therefore, the simple classification of land cover derived from satellite images was adopted in this study, and could indirectly reflect soil moisture content. Finally, multiple regression analyses were performed on the wind velocity, temperature, humidity, and bare land area of river dust episodes. The relationships between these factors and the river dust episodes were analyzed to examine which factors caused the episodes. Results of this study can serve as a reference for river dust management.

### Highlights

- Most river dust has a particle size of PM_10_. The relationship between PM_10_ concentration and meteorological factors or bare land area can be used to construct an early warning system for river dust episodes.
- Air quality monitoring stations are typically not located in areas with river dust emission. Therefore, a time lag effect existed between meteorological factors and PM_10_ concentration. This effect can be used for early warnings of river dust episodes.
- In early winter, a positive correlation existed between bare land area and PM_10_ concentrations, but this relationship was not statistically significant in late winter. The primary reason is that the coarse earth and sand particles of the bare land create a protective armoring that decreases PM_10_ concentrations.

## 2. Materials and Methods

### 2.1. Study Area

**Figure 1:**
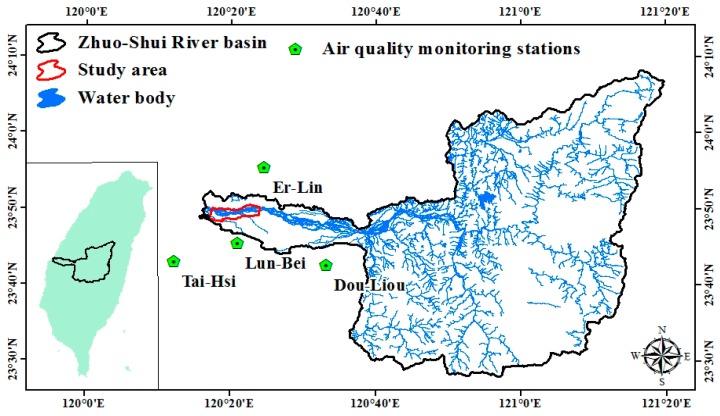

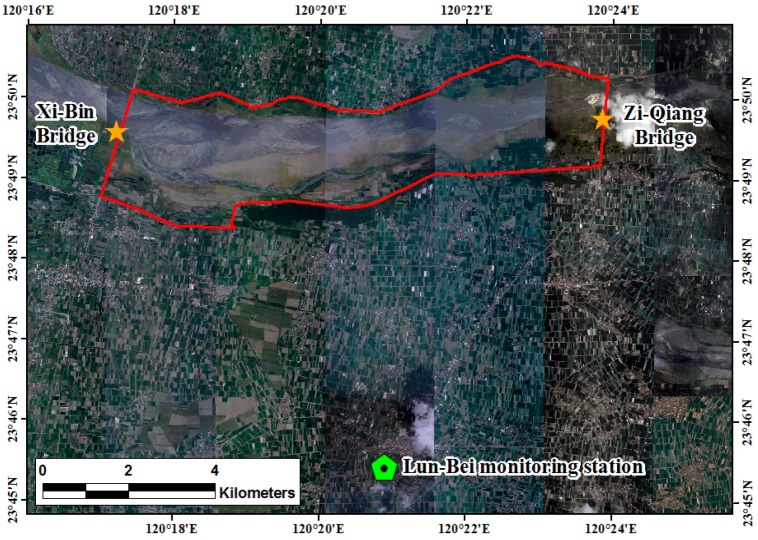
Location of study area and air quality monitoring stations.

**Figure 2:**
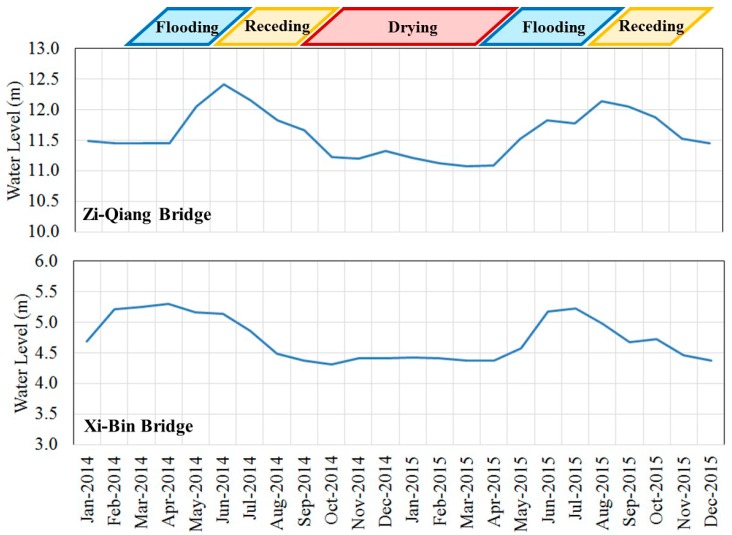
The seasonal variations of water level at Zi-Qiang Bridge and Xi-Bin Bridge.

The Zhuo-Shui River is located in central Taiwan. It consists of layers of shale and sandstone that are vulnerable to erosion. It frequently suffers from rain erosion. A large amount of sediment is transported by the stream. All year round it is muddy, which is also the origin of its name. In the outlet of the Zhuo-Shui River, due to congenital conditions, soil particle sizes are extremely small and their binding strengths are weak. The soil is struck by the northeast monsoon every dry season. Thus, the streets on the southern side of the river are dusty and the safety of both lives and property is threatened. Hence, the outlet of the Zhuo-Shui River (where there is potential for dust hazards during the dry seasons) is selected as the study area (Figure 1). Its range is from Zi-Qiang Bridge to Xi-Bin Bridge. Annually, the flooding–receding process occurs during March to October, which is the wet season, whereas the rest of the year is the dry season (Figure 2). The land is used to grow watermelons. The floodplain is dominated by growth wild sugarcane grasses. The areas near the river embankment have been cultivated to grow melons, peanuts, vegetables, and other crops [35].

### 2.2. Materials

**Figure 3:**
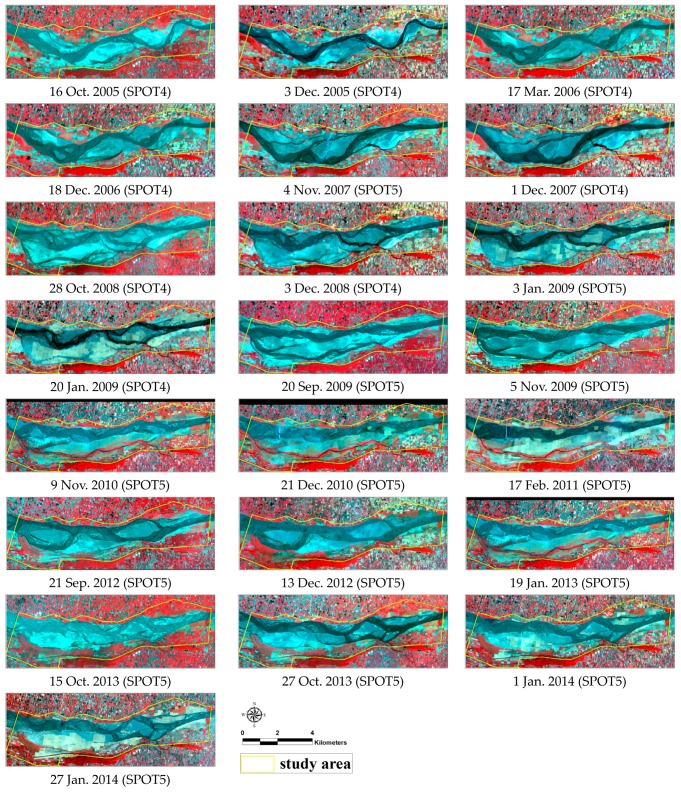
Satellite images used in this study.

Out of the four air quality monitoring stations around the research area, only the meteorological data of the Lun-Bei monitoring station were selected to construct the model due to its proximity to the southern bank of the river and its location in Lun-Bei village, which is subject to river dust under northeast monsoons. PM_10_, PM_2.5_, rainfall, wind velocity, wind direction, temperature, and relative humidity data from 2005 to 2014 were analyzed in this study. For more information regarding the monitoring devices and placement please refer to [36]. The National Central University Center for Space and Remote Sensing Research’s Satellite pour l’observation de la Terre (SPOT) obtained 22 cloud-free SPOT4/5 images (Figure 3) in which land cover data were extracted from those images. In order to reflect the status of land cover for each river dust episode, the date of the image should be selected in proximity to the episode. The date difference between each episode and the corresponding image was limited to be within 15 days of the cloud-free image acquisition. 

### 2.3. Methods

#### 2.3.1. Screening for Days with River Dust Episodes

**Figure 4:**
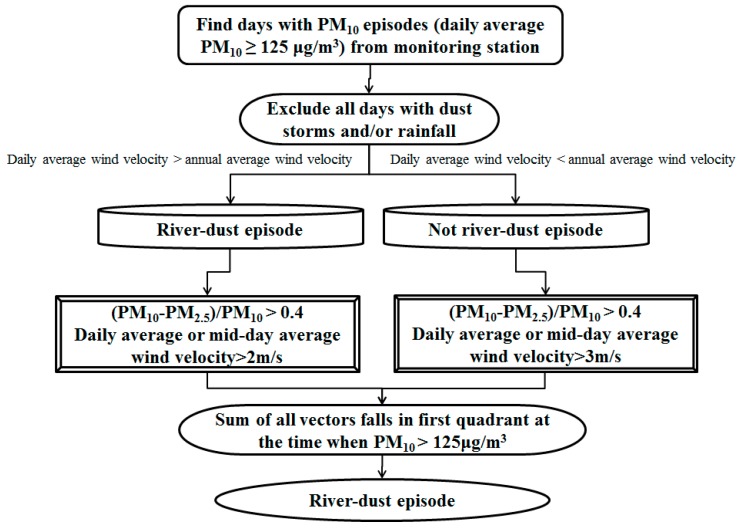
Process to screen for days with river dust episodes.

According to Taiwan’s Environmental Protection Administration, Executive Yuan [37], a PM_10_ concentration over 125 μg/m^3^ can affect human health. The long-distance transportation of dust storms from China should be removed first. The other events, the non-long distance transportation, are considered local dust events. The local dust events include river dust, detention-dust, and agriculture waste burning events. Only river dust has a positive relation with wind speed; the others have a negative relation with wind speed. Therefore, the river dust event can be screened by the threshold of wind speed and the ratio of (PM_10_−PM_2.5_)/PM_10_ [38]. The steps to screen for days with river dust episodes are as follows (Figure 4):

① From monitoring station logs, find days with PM_10_ episodes (defined as days when the average PM_10_ concentration was greater than or equal to 125 μg/m^3^).
② Exclude all days with dust storms and/or rainfall.
③ Obtain the annual average wind velocity and the daily average wind velocity for all remaining days. For a given day, if the daily average wind velocity is higher than the annual average wind velocity, then that day is temporarily defined as being a river dust episode.
④ For a day temporarily defined as being a river dust episode, if the ratio of (PM_10_−PM_2.5_)/PM_10_ is greater than 0.4, and either the daily average wind velocity or the mid-day (12:00–17:00) average wind velocity is greater than 2 m/s, then that day is confirmed as being a river dust episode.
⑤ For all remaining days, if the ratio of (PM_10_−PM_2.5_)/PM_10_ is greater than 0.4, and either the daily average wind velocity or the mid-day (12:00–17:00) average wind velocity is greater than 3 m/s, then that day is redefined as being a river dust episode.
⑥ River dust episodes mostly occur during the northeastern monsoon season and the Lun-Bei Station is located slightly to the west of the south bank of the Zhuo-Shui River. Thus, the dust primarily comes from the northeastern direction. Among the above results, days without a northeastern wind need to be excluded. Wind direction is defined as the sum of all wind vectors at the time when PM_10_ is greater than 125 μg/m^3^. If the result falls in the first quadrant, then the wind is defined as a northeastern wind.

#### 2.3.2. Image Classification

**Figure 5:**
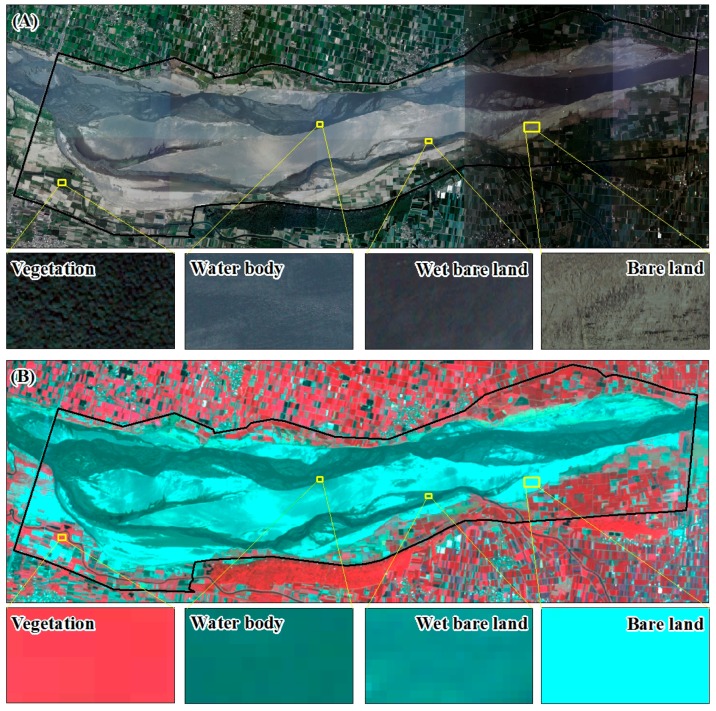
Training samples (**A**) aerial photo (**B**) satellite image (false color composite).

**Figure 6:**
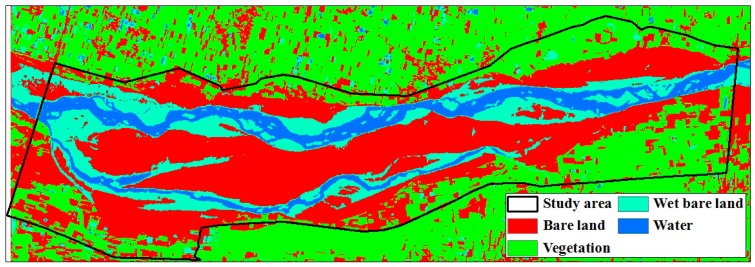
Result of land cover classification.

To derive the potential dust emission zones at the estuary of the Zhuo-Shui River, image classification was performed using a supervised classification method and a maximum likelihood algorithm. In a supervised classification scheme, training samples that are representative of the land cover classes of interest should be selected [39]. In this study, four types of land cover (*i.e.*, vegetation, water, wet bare land, and bare land) were selected from the research area at the lower reaches of the river between the dikes from the Zi-Qiang Bridge to the Xi-Bin Bridge (Figure 5 and Figure 6). The probability distribution was calculated using a maximum likelihood algorithm; in particular, a multivariate normal distribution is assumed as the probability density function for each land cover class. Each pixel is then evaluated and assigned to the class that has the highest probability [40].

#### 2.3.3. Accuracy Assessment of Kappa Coefficient

The kappa coefficient is used to compare the results of image classification with the results of completely random classification and is the reduction in percent error from using a classification method. In other words, the kappa coefficient acts as an indicator of the difference in magnitude of accuracy between the results of a classification method and random classification. The kappa coefficient is calculated from reciprocal operations within an error matrix. It incorporates both commission and omission errors and was later widely applied to assess the accuracy of remotely sensed image classifications. Generally, the kappa coefficient has a value between 0 and 1; a higher kappa coefficient represents greater accuracy in the classification. The formula is as follows [41]:

(1) $$\mathrm{kappa}\mathrm{coefficient}=\frac{N\sum_{i=1}^{n}X_{ii}-\sum_{i=1}^{n}\left(X_{i+}\times X_{+i}\right)}{N^{2}-\sum_{i=1}^{n}\left(X_{i+}\times X_{+i}\right)}$$

where *n* is the number of rows in the matrix, ***X_ii_*** is the number of samples in a diagonal line across the rows and columns of the classification matrix, ***X_i+_***, ***X_+i_*** are the number of samples in each row and each column of the classification matrix, and *N* is the total number of samples.

#### 2.3.4. Multiple Regression Analysis

The analysis of a single explanatory variable and its correlation to a single variable is called a simple regression analysis and is expressed by y = f(x); the primary principle behind the calculation is the method of least squares. When an analysis requires multiple explanatory variables, it is called a multiple regression analysis. This model can be expressed as follows:

(2) $$Y=\beta_{0}+\beta_{1}X_{1}+\beta_{2}X_{2}+\cdot \cdot \cdot +\beta_{n}X_{n}$$

where Y is the dependent variable, *X*_i_ is the explanatory variable, and *β*_i_ is the regression coefficient. The model can be rewritten as a matrix, as follows:

(3) $$\left[\begin{array}{l} Y_{1}\\ Y_{2}\\ \;\;\vdots \\ Y_{n} \end{array}\right]=\left[\begin{array}{l} 1\;\;\;\;\;\;X_{11}\;\;\;\;\cdots \;\;\;\;\;X_{n1}\\ 1\;\;\;\;\;\;X_{12}\;\;\;\;\cdots \;\;\;\;\;X_{n2}\\ \vdots \;\;\;\;\;\;\;\vdots \;\;\;\;\;\;\;\;\;\;\ddots \;\;\;\;\;\;\;\vdots \\ 1\;\;\;\;\;\;X_{1n}\;\;\;\;\cdots \;\;\;\;\;X_{nn} \end{array}\right]\left[\begin{array}{l} \beta_{0}\\ \beta_{1}\\ \;\vdots \\ \beta_{n} \end{array}\right]$$

Stepwise regression was chosen as the method for the multiple regression analyses in this study. Stepwise regression selects a new set of independent variables to construct a regression model with a better fit by retaining independent variables that have a significant correlation with the dependent variables and deleting those that do not, thereby reducing unnecessary independent variables and the model’s prediction parameters. The selection of variables is usually based on the statistical significance of an F-test and the selection standard for independent variable values is their effects on the model. River dust warnings, like weather forecasts, should be provided on a daily basis to conform to human habit. Therefore, multiple regression analyses were performed on the factors that affect river dust episodes and the daily average PM_10_ or the daily maximum PM_10_ to explore the relationships between each of the factors and river dust episodes.

## 3. Results

### 3.1. Relationships between PM_10_ Concentration and Meteorological Factors

Analyzing data from the Lun-Bei air quality monitoring station resulted in the designation of 74 days between 2005 and 2014 as being river dust episodes. These results were divided into subsets for constructing and verifying the model. Data from 57 days from 2005 to 2010 (77%) were used for model construction and 17 days from 2011 to 2014 (23%) were used for model validation.

**Figure 7:**
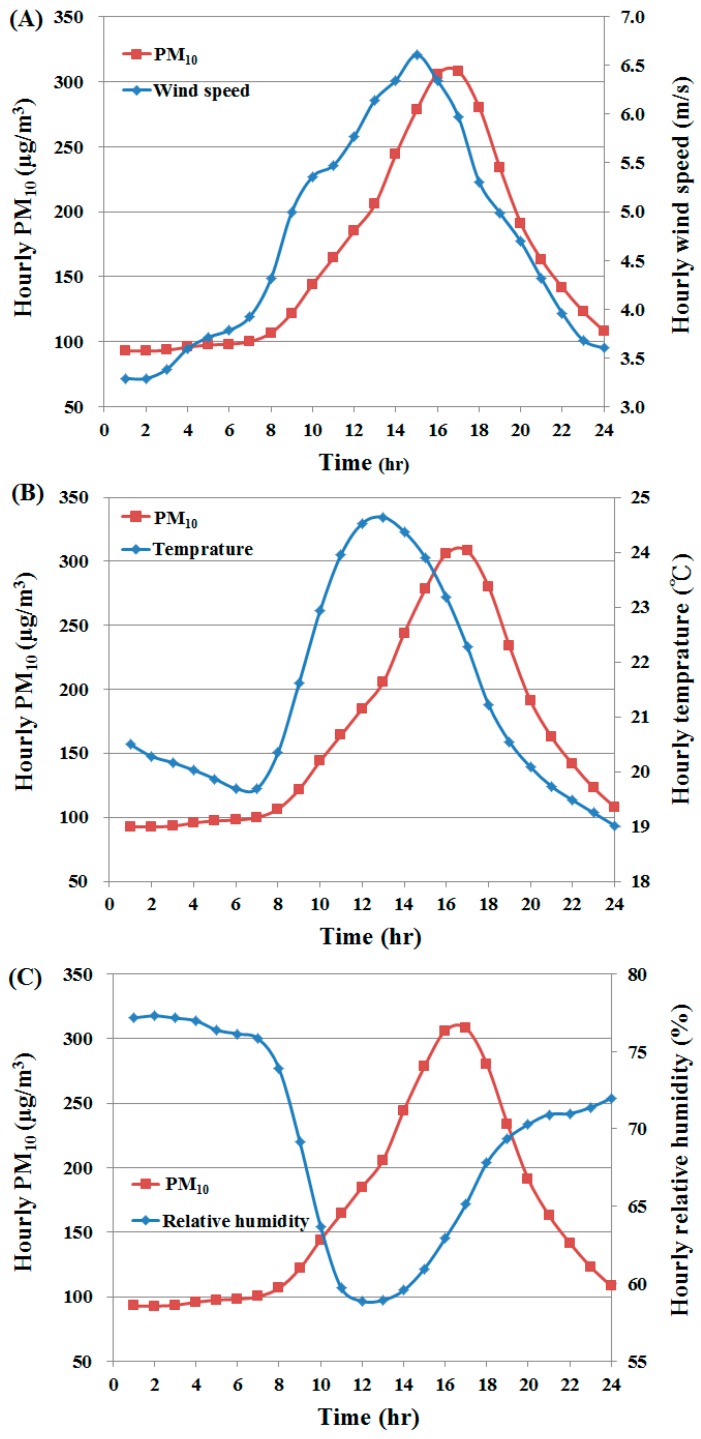
Hourly PM_10_ for a day of river dust occurrence with corresponding meteorological data: (**A**) wind velocity; (**B**) temperature; (**C**) relative humidity.

Examination of the relationship between daily variations in PM_10_ and the immediate meteorological factors in the 57 days with river dust episodes showed a time lag effect ranging from 2 to 5 h for wind velocity, temperature, and relative humidity (Figure 7). Therefore, all subsequent regression analyses used meteorological values from 2 h prior to the time of the daily maximum PM_10_.

**Figure 8:**
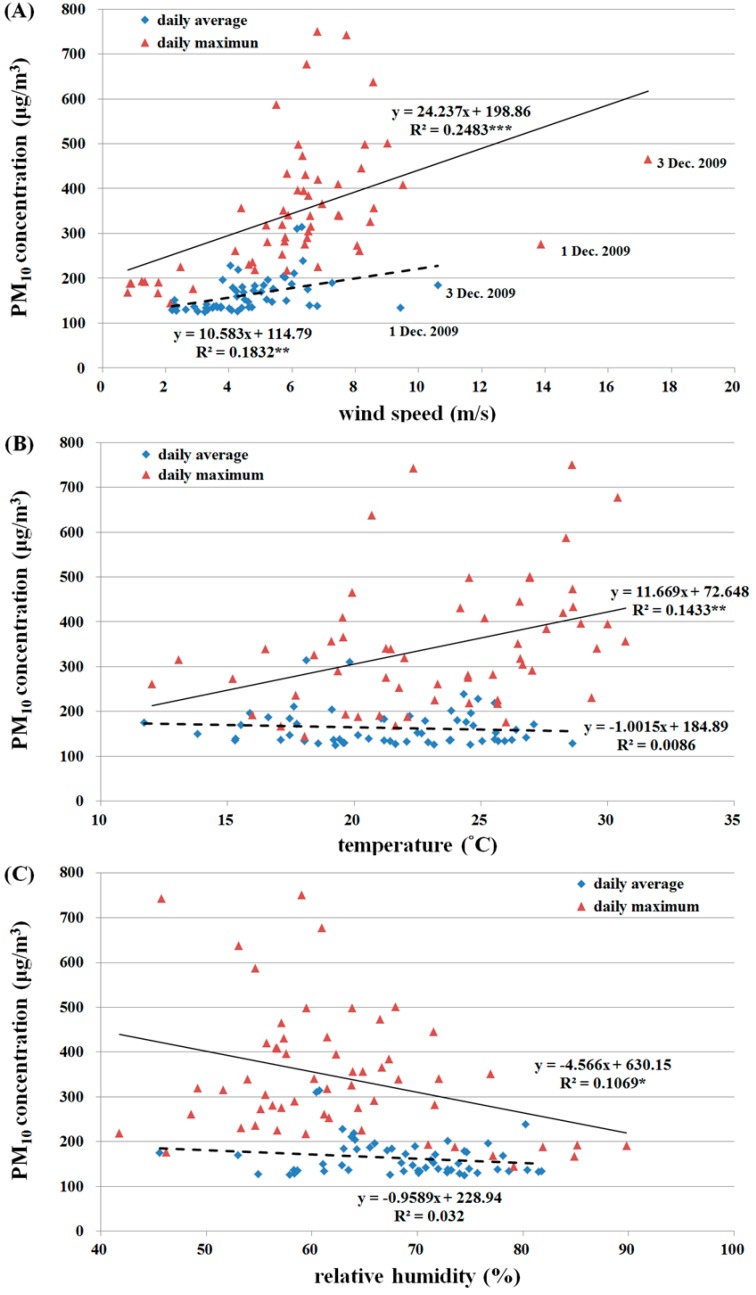
Relationship between daily average/maximum PM_10_ and (**A**) wind speed; (**B**) temperature; and (**C**) relative humidity. Dashed lines are regression lines of daily average PM_10_ and bold lines are regression lines of daily maximum PM_10_.

In particular, the wind velocities on 1 December 2009 and 3 December 2009 were substantially higher than those on all other days, but neither the daily average nor the daily maximum PM_10_ levels increased substantially on those two dates. Clearly, the higher wind velocity was conducive to the dispersion of suspended particles. Temperature and relative humidity did not seem to be correlated to the daily average or the daily maximum PM_10_ (Figure 8).

### 3.2. Relationships between PM_10_ Concentration and Bare Land Area

**Figure 9:**
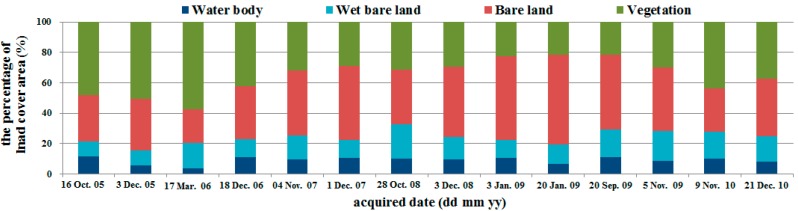
Land cover changes in the study area from 2005 to 2010 (model establishment).

**Figure 10:**
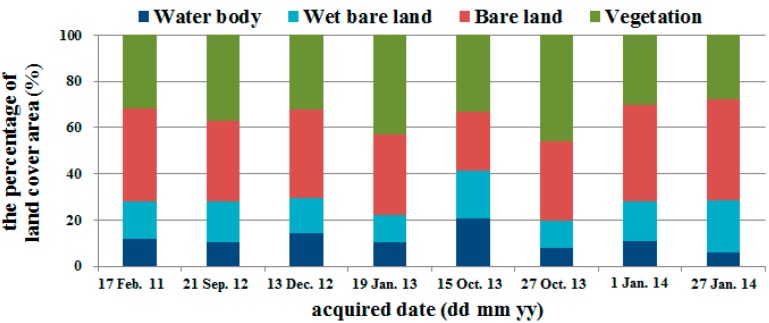
Land cover changes in the study area from 2011 to 2014 (model validation).

**Figure 11:**
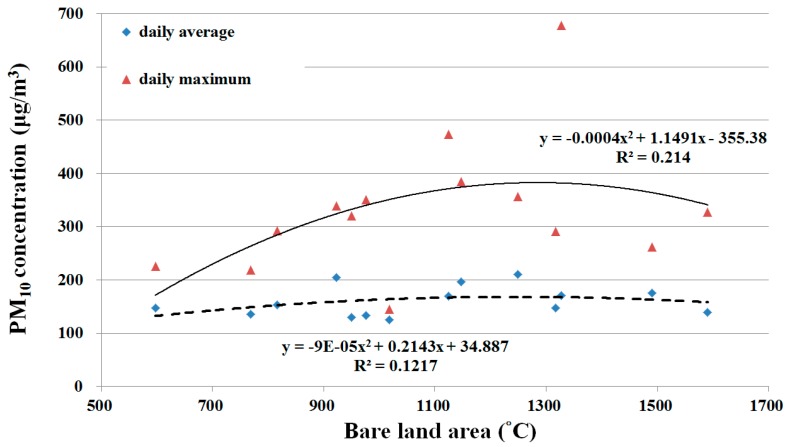
Relationship between daily average/maximum PM_10_ and bare land area. Dashed line is regression line of daily average PM_10_ and bold line is regression line of daily maximum PM_10_.

Twenty-two satellite images were chosen that were taken closest in time to the 74 days of river dust episodes and did not have cloud cover. In coordination with the meteorological data models, 14 satellite images from 2005 to 2010 were used for model construction and eight satellite images from 2011 to 2014 were used for model validation. Image classification results were considered highly accurate if the Kappa coefficient was larger than 0.7. The water-covered land is the primary location of the river channel. After waters recede during the winter drought season, the area of the water-covered land gradually decreases. The wet bare land, because it is adjacent to water-covered land, has a higher moisture content and smaller dirt particles. These two types of land comprised a relatively small proportion of the entire research area and the sizes of their areas did not vary greatly during the research time period. Bare land and vegetation-covered land were affected by fluctuations of the primary river channel, and there was relatively greater variation in the sizes of their areas (Figure 9 and Figure 10). Regression analyses were performed on the 14 satellite images used for model construction and PM_10_. Results showed a quadratic relationship between daily average and maximum PM_10_ and bare land area, but the coefficients of determination were low at 0.1217 and 0.214 (Figure 11). However, an examination of the trends showed that river dust amounts increased as the bare land area increased, but as the bare land area increased because of receding waters, the PM_10_ concentration did not continue to increase. On-site observations revealed that after several strong wind events, the proportion of coarse earth and sand particles increased, which produced a protective armoring effect.

## 4. Discussion

On days with river dust episodes, the correlations between daily average PM_10_ or daily maximum PM_10_ and temperature or relative humidity were not significant. It was speculated that spatial and temporal variations in temperature and relative humidity are complex and readily affected by land cover type, season, wind direction, and rainfall. Thus, the effects of these two meteorological factors were diluted in the analysis of the daily average PM_10_ and unable to reflect variations in PM_10_ concentrations. A summary of the correlations between PM_10_ levels on both temporal scales and meteorological factors (Table 1) showed that for all temporal scales, the highest correlation was found for wind velocity and the next highest correlation for temperature; relative humidity was excluded in the stepwise regression.

The relationships between hourly PM_10_ levels and hourly fluctuations in wind velocity, temperature, and relative humidity showed a time lag effect between peak values of PM_10_ and each of the meteorological factors. To incorporate this effect in the river dust amount estimation model, regression analysis was performed on meteorological values from 2 h prior to the daily maximum PM_10_; doing so increased the coefficient of determination for the daily maximum PM_10_ model to 0.394. A practical application of this finding is that meteorological data can be used to predict the PM_10_ concentration 2 h later and are highly suitable for use as a warning.

In addition, including the bare land area in the multiple regression analysis showed that all factors for predicting daily average PM_10_ were excluded by the stepwise analysis process. For predicting the daily maximum PM_10_, the coefficient of determination was as high as 0.67. Therefore, bare land area was the key factor affecting PM_10_ concentration.

## 5. Validation

The constructed prediction model was verified using data from river dust episodes from 2011 to 2014. Meteorological factors (Table 2) and bare land area (Table 3) were verified and examined individually.

When only meteorological factors were included in the prediction model, the coefficient of determination for daily average PM_10_ was 0.202 and the error of its predictive power was approximately 80%. However, the validation results in Table 2 show that the highest percent error was only 39%. Thus, the model was able to predict daily average PM_10_ even though the coefficient of determination was relatively low. The coefficient of determination for daily maximum PM_10_ was 0.394 and the error of its predictive power was approximately 60%. Validation results in Table 2 show that only the errors from 9 February 2011 and 18 January 2014 were relatively high. When three variables are included in the prediction model, the coefficient of determination for daily maximum PM_10_ was 0.67 and the error of its predictive power was approximately 33%. Validation results in Table 3 show that only the errors from 9 February 2011 and 18 January 2014 were higher than 33%. This confirms that the model constructed in this study was able to effectively predict the river dust amount on days with river dust episodes. However, beginning in January of the following year, the fine particles of earth and sand on the bare land had already blown away, leaving only the coarse particles that formed a protective armoring. Thus, the size of surface particles became a factor in the PM_10_ concentration on bare land. This dynamic variation in the size of surface particles on bare land can be examined by future studies and can serve to enhance the accuracy of prediction models. 

## 6. Conclusions

The problem of river dust at Taiwan’s rivers and river estuary has begun to receive attention in recent years. Therefore, it is extremely important to examine factors that affect river dust occurrence and provide this information to river management authorities so they can devise river dust control policies as soon as possible. The factors and selection of river dust episodes used in this study are based on statistical results. By analyzing the relationship between PM_10_ and meteorological factors or bare land area on days with river dust episodes, a clear time lag effect was found between meteorological factors and hourly PM_10_ concentrations. This characteristic can be used to construct a model to predict the PM_10_ concentrations in the subsequent 2 h and to provide early warning for local residents to adopt appropriate protective measures. No single factor had adequate power to explain daily average or daily maximum PM_10_ concentration. Including multiple factors in stepwise regression analysis showed that although the correlation between meteorological factors and daily average PM_10_ concentration was significant, the coefficient of determination was only 0.202; the correlation with daily maximum PM_10_ was also significant and the coefficient of determination was 0.394. Including both meteorological factors and bare land area, the coefficient of determination for daily maximum PM_10_ concentration was as high as 0.67 and the correlation was significant. Validation results showed that the model could effectively predict the PM_10_ concentration, but beginning in January of the following year, the fine particles of earth and sand on the bare land had already blown away, leaving only coarse particles that formed a protective armoring. Therefore, the PM_10_ concentration did not increase markedly thereafter. Nevertheless, local differences in land cover conditions of bare land, such as particle size and moisture content, also have an effect on river dust emissions. It is suggested that future studies analyze regional microsurface conditions of the riverbed, using remote sensing technology to determine the particle size distribution of riverbed materials and more accurately determine potential zones of river dust emissions. This would allow for more effective prediction, management, and prevention of river dust episodes.

## Figures and Tables

**Table 1:** Estimations model of PM_10_ concentration on different temporal scales (W_D_: daily average wind speed, W_H-2h_: wind velocity 2 h prior, T_H-2h_: temperature 2 h prior, A_bare_: bare land area).

Model	Items	Number of Records	Equations	R^2^
1	Daily Average	57	PM_10_ = 11.82 W_D_ + 110.46	0.202 ***
2	Daily Maximum	57	PM_10_ = 24.37 W_H-2h_ + 11.78 T_H-2h_ − 76.63	0.394 ***
3	Daily Maximum	14	PM_10_ = 35.69 W_H-2h_ + 17.62 T_H-2h_ + 0.08A_bare_ − 363.98	0.67 **

Notes: ** *p* < 0.01, *** *p* < 0.001.

**Table 2:** Validation of meteorological factors with daily average PM_10_ or daily maximum PM_10_.

Date (dd mm yy)	Daily Average PM_10_ Validation			Daily Maximum PM_10_ Validation		
	Actual PM_10_	Predicted PM_10_	Percent Error	Actual PM_10_	Predicted PM_10_	Percent Error
9 Feb. 2011	133	147	10.59	188	312	65.75
14 Sep. 2012	190	163	−14.08	461	467	1.24
15 Sep. 2012	151	179	18.94	425	457	7.62
28 Sep. 2012	272	198	−27.23	548	432	−21.20
23 Dec. 2012	135	189	39.42	318	307	−3.42
17 Jan. 2013	153	178	16.28	358	355	−0.71
6 Mar. 2013	125	132	5.77	158	165	4.18
3 Oct. 2013	147	161	9.45	253	352	39.06
5 Oct. 2013	167	171	2.15	497	448	−9.85
23 Oct. 2013	141	170	20.10	375	431	15.04
24 Oct. 2013	207	179	−13.61	471	413	−12.37
25 Oct. 2013	185	170	−8.31	342	331	−3.19
27 Dec. 2013	230	184	−19.96	355	266	−25.15
4 Jan. 2014	164	155	−5.61	266	283	6.46
5 Jan. 2014	159	148	−6.94	203	185	−8.92
14 Jan. 2014	141	185	31.39	371	340	−8.24
18 Jan. 2014	263	184	−30.16	680	319	−53.16

**Table 3:** Validation of daily maximum PM_10_ with bare land area.

Date (dd mm yy)	Date of Satellite Image (dd mm yy)	Actual PM_10_	Predicted PM_10_	Percent Error
9 Feb. 2011	17 Feb. 2011	188	298	58.42
28 Sep. 2012	21 Sep. 2012	548	464	−15.31
23 Dec. 2012	13 Dec. 2012	318	285	−10.48
17 Jan. 2013	19 Jan. 2013	358	348	−2.66
5 Oct. 2013	15 Oct. 2013	497	468	−5.77
25 Oct. 2013	27 Oct. 2013	342	314	−8.15
4 Jan. 2014	1 Jan. 2014	266	259	−2.55
18 Jan. 2014	27 Jan. 2014	680	314	−53.85
